# Berberine Antifungal Activity in Fluconazole-Resistant Pathogenic Yeasts: Action Mechanism Evaluated by Flow Cytometry and Biofilm Growth Inhibition in Candida spp.

**DOI:** 10.1128/AAC.01846-15

**Published:** 2016-05-23

**Authors:** Anderson Ramos da Silva, João Batista de Andrade Neto, Cecília Rocha da Silva, Rosana de Sousa Campos, Rose Anny Costa Silva, Daniel Domingues Freitas, Francisca Bruna Stefany Aires do Nascimento, Larissa Nara Dantas de Andrade, Letícia Serpa Sampaio, Thalles Barbosa Grangeiro, Hemerson Iury Ferreira Magalhães, Bruno Coêlho Cavalcanti, Manoel Odorico de Moraes, Hélio Vitoriano Nobre Júnior

**Affiliations:** a School of Pharmacy, Laboratory of Bioprospection and Experiments in Yeast (LABEL), Federal University of Ceara, Fortaleza, CE, Brazil; b Department of Biology, Science Center, Molecular Genetics Laboratory, Federal University of Ceara, Fortaleza, CE, Brazil; c School of Pharmacy, Federal University of Paraiba, João Pessoa, PB, Brazil; d Department of Physiology and Pharmacology, Federal University of Ceara, Fortaleza, CE, Brazil

## Abstract

The incidence of fungal infections and, in particular, the incidence of fungal antibiotic resistance, which is associated with biofilm formation, have significantly increased, contributing to morbidity and mortality. Thus, new therapeutic strategies need to be developed. In this context, natural products have emerged as a major source of possible antifungal agents. Berberine is a protoberberine-type isoquinoline alkaloid isolated from the roots, rhizomes, and stem bark of natural herbs, such as Berberis aquifolium, Berberis vulgaris, Berberis aristata, and Hydrastis canadensis, and of Phellodendron amurense. Berberine has been proven to have broad antibacterial and antifungal activity. In the present study, the potential antifungal effect of berberine against fluconazole-resistant Candida and Cryptococcus neoformans strains, as well as against the biofilm form of Candida spp., was assessed. The antifungal effect of berberine was determined by a broth microdilution method (the M27-A3 method of the Clinical and Laboratory Standards Institute) and flow cytometry techniques, in which the probable mechanism of action of the compound was also assessed. For biofilm assessment, a colorimetric 3-(4,5-dimethyl-2-thiazolyl)-2,5-diphenyl-2H-tetrazolium bromide (MTT) assay was used to determine the susceptibility of sessile cells. The isolates used in the study belonged to the Laboratory of Bioprospection and Experiments in Yeast (LABEL) of the Federal University of Ceará. After 24 and 72 h, fluconazole-resistant Candida and Cryptococcus neoformans strains showed berberine MICs equal to 8 μg/ml and 16 μg/ml, respectively. Cytometric analysis showed that treatment with berberine caused alterations to the integrity of the plasma and mitochondrial membranes and DNA damage, which led to cell death, probably by apoptosis. Assessment of biofilm-forming isolates after treatment showed statistically significant reductions in biofilm cell activity (*P* < 0.001).

## INTRODUCTION

Yeasts are the most common agents in opportunistic fungal infections, which mainly affect immunocompromised patients (1). Candida yeasts are pathogens that appear in nosocomial infections. Candida spp. are the third most commonly isolated agents of fungal infections acquired in hospitals (2) and are noted for their significant prevalence in different medical centers and for the complications that they cause. In addition, these infections are difficult to diagnose, leading to mortality rates of 50% (3, 4).

The opportunistic and pathogenic fungus Cryptococcus neoformans can cause life-threatening meningitis. Each year, it is estimated that about 1 million new cases of cryptococcal meningitis occur throughout the world, making C. neoformans infection a global public health concern (5, 6). Although antifungal drugs for the treatment cryptococcosis are available, they often fail for several reasons, especially because of C. neoformans resistance to the antifungal agents (7).

Another current problem are nosocomial infections associated with biofilms. These infections are difficult to treat because these microbial structures are resistant both to the activity of conventional antimicrobial agents and to host defense mechanisms. Candida spp. are among the main organisms responsible for these infections, which are mainly associated with implanted medical devices (2).

In this context, herbal antifungal agents have gained importance due to their natural origin (8). Berberine is a protoberberine-type isoquinoline alkaloid isolated from the roots, rhizomes, and stem bark of natural herbs, such as Berberis aquifolium, Berberis vulgaris, Berberis aristata, Hydrastis canadensis, Phellodendron amurense, Coptis chinensis, and Tinospora cordifolia (9–11). In some studies, it was shown that berberine extract has significant antimicrobial activity against bacteria, viruses, protozoa, fungi, and yeasts (12).

Studies have also shown that berberine has a significant antifungal effect against Candida albicans strains (13–15).

The aim of the present study was to evaluate the antifungal potential of berberine against different fluconazole-resistant Candida and Cryptococcus neoformans strains. In addition, the effect of berberine against C. albicans and its activity against biofilms produced by Candida tropicalis were assessed through flow cytometry procedures and a standard single cell gel electrophoresis (comet) assay.

## MATERIALS AND METHODS

### Isolates.

Eleven Candida strains that were isolated from blood samples at the Central Public Health Laboratory (LACEN-CE) and that are part of the Yeast Collection from the Laboratory of Bioprospection and Experiments in Yeast, affiliated with the School of Pharmacy, Federal University of Ceara (LABEL/FF/UFC), and two ATCC strains (Candida parapsilosis ATCC 22019 and Candida krusei ATCC 6258) were used. The strains were inoculated in Sabouraud dextrose agar (HiMedia, Mumbai, India) and incubated at 37°C for 24 h. Then, the strains were grown in CHROMagar Candida (HiMedia), in order to evaluate their purity.

### Molecular identification.

Genomic DNA was purified using a protocol based on the cetyltrimethylammonium bromide (CTAB) surfactant, as previously described (16). The nuclear DNA region comprising internal transcribed spacer 1 (ITS1), ITS2, and the 5.8S rRNA gene was amplified by PCR using primers ITS4 (5′-TCCTCCGCTTATTGATATGC-3′) and ITS5 (5′-GCAAGTAAAAGTCGTAACAAGA-3′), as suggested by White et al. (17). Amplification reactions were performed in a final volume of 25 μl, which contained genomic DNA (300 to 400 ng), 1× GoTaq reaction buffer (Promega, Madison, WI, USA), 1.5 mM MgCl_2_, 200 μM each deoxynucleoside triphosphate (GE Healthcare Life Sciences, Piscataway, NJ, USA), 0.5 μM each primer, and 1.25 U of GoTaq DNA polymerase (Promega). Reactions were carried out in a Mastercycler gradient thermocycler (Eppendorf, Hamburg, Germany) programmed for an initial denaturation step (2 min at 95°C), followed by 35 cycles of 1 min at 95°C, 1 min at 60°C, and 3 min at 72°C. The last cycle was followed by a final incubation of 10 min at 72°C. Control samples containing all components of the reaction mixture except DNA were used to test that no DNA contamination occurred. Amplification specificity was determined by 1.0% agarose gel electrophoresis (18). The remaining amplified products were purified using an Illustra GFX PCR DNA and gel band purification kit (GE Healthcare Life Sciences). The concentrations of the purified amplicons were determined by measuring the absorbance of 10-fold dilutions at 260 nm. DNA sequencing was performed at Macrogen Inc. (Seoul, South Korea) using the Sanger chain-termination method. Both strands of each amplicon were sequenced using primers ITS4 and ITS5. Subsequently, the sequences were assembled using the Phred/Phrap/Consed package (19–21). The start and end of the ITS1 and ITS2 sequences were identified by comparison to annotated sequences from the ITSoneDB (22) and ITS2 (23) databases, respectively. The ITS/5.8S sequences were deposited in the GenBank database and compared to those available in public DNA sequence databases using the BLAST program (24).

### *In vitro* antifungal activity.

The broth microdilution (BMD) susceptibility test was conducted according to the guidelines in document M27-A3 of the Clinical and Laboratory Standards Institute (CLSI) (25), using RPMI 1640 broth (pH 7.0) buffered with 0.165 M MOPS (morpholinepropanesulfonic acid) (Sigma Chemical, St. Louis, MO). Fluconazole (FLC; Merck Sharp & Dohme, São Paulo, Brazil) was dissolved in distilled water, and the berberine chloride solution (Sigma Chemical) was prepared in dimethyl sulfoxide (DMSO; Sigma Chemical). FLC and berberine were tested at concentrations ranging from 0.125 to 64 μg/ml. The yeasts and compounds were incubated in 96-well culture plates at 35°C for 24 h, and the results were examined visually, as recommended by CLSI (26). The MIC of each compound was determined as the concentration that inhibited 50% of fungal growth. Cutoff points were determined according to the information in CLSI document M27-S4 (26). Strains C. parapsilosis ATCC 22019 and C. krusei ATCC 6258 were used as controls (25).

### Mechanism-of-action studies.

In order to assess cell density, membrane integrity, mitochondrial transmembrane potential (Δψm), and DNA damage (comet assay), the fluconazole-resistant C. albicans 2 strain was exposed for 24 h to increasing berberine concentrations (MIC, 2× MIC, and 4× MIC). All tests were conducted in triplicate in three independent experiments.

### Yeast suspension preparation.

Cell suspensions were prepared from cultures in the exponential growth phase. Cells were collected by centrifugation (1,600 × *g* for 10 min at 4°C) and washed twice with a 0.85% saline solution by centrifugation at 1,200 × *g* for 5 min at 4°C. Subsequently, they were resuspended (∼10^6^ cells/ml) in HEPES buffer (pH 7.2) supplemented with 2% glucose. Amphotericin B (AMB; 4 μg/ml; Sigma Chemical) was used as a cell death control (27, 28).

### Cell density and membrane integrity determination.

Fungal strain cell density and membrane integrity were evaluated by excluding 2 mg/liter propidium iodide (PI). Aliquots from yeasts incubated for 24 h with berberine, FLC, and AMB were analyzed using flow cytometry. A total of 10,000 events were evaluated per experiment (*n* = 2), and cellular debris was omitted from the analysis. Cell fluorescence was determined by flow cytometry using a Guava EasyCyte minisystem cytometer (Guava Technologies Inc., Hayward, CA, USA), and the results were analyzed using CytoSoft (version 4.1) software (29, 30).

### Measurement of Δψm.

Δψm was determined by measuring rhodamine 123 dye retention by the mitochondria of yeast cells after 24 h of exposure. Cells were washed with phosphate-buffered saline (PBS), incubated with 5 mg/liter rhodamine 123 at 37°C for 30 min in the dark, and then washed twice with PBS. Fluorescence was measured by flow cytometry (Guava EasyCyte minisystem). A total of 10,000 events were evaluated per experiment (*n* = 2), and cellular debris was omitted from the analysis (27, 30).

### Yeast alkaline comet assay.

An alkaline comet assay was conducted essentially as described by Miloshev et al. (31). Up to 200 μl of 0.5% agarose was spread on each slide, and this supportive agarose layer was air dried prior to application of the cell suspension. C. albicans 2 cells were collected by centrifugation in an Eppendorf microcentrifuge for 5 min. Then, the cells were washed with distilled water and resuspended in S buffer (1 M sorbitol, 25 mM KH_2_PO_4_, pH 6.5). Aliquots of approximately 5 × 10^4^ cells were mixed with 0.7% low-melting-point agarose containing 2 mg/ml Zymolyase 20T (Seikagaku Corp., Japan), and the cells were subsequently spread over the slides. The slides were then covered with coverslips and incubated for 20 min at 30°C. In order to minimize endogenous cell enzyme activity, all further procedures were performed in a cold room at 8 to 10°C. The coverslips were removed, and the slides were incubated in 30 mM NaOH, 1 M NaCl, 0.1% laurylsarcosine, and 50 mM EDTA (pH 12.3) for 1 h, in order to lyse the spheroplasts. The slides were rinsed three times for 20 min each time in 30 mM NaOH and 10 mM EDTA (pH 12.4), in order to unwind the DNA. Subsequently, the slides were subjected to electrophoresis in the same buffer. Electrophoresis was performed for 20 min at 0.5 V/cm and 24 mA. After electrophoresis, the gels were neutralized by submerging the slides in 10 mM Tris-HCl, pH 7.5, for 10 min, followed by consecutive 10-min incubations in 76% and 96% ethanol. Finally, the slides were left to air dry, stained with ethidium bromide (1 mg/ml), and visualized by fluorescence microscopy (32). All steps mentioned above were conducted in the dark, in order to prevent additional DNA damage. Images of 100 randomly selected cells (50 cells from each of the two replicate slides) were analyzed for each experimental group. Cells were visually scored and assigned to one of five classes according to tail size (from undamaged [class 0] to maximally damaged [class 4]), and a damage index value was calculated for each cell sample. The damage index values therefore ranged from 0 (completely undamaged, 100 cells × 0) to 400 (maximum damage, 100 cells × 4) (33). The frequency of cells with tails, which was considered an indicator of DNA damage, was calculated on the basis of the number of cells with tails (DNA strand breaks) and the number of cells without tails (28, 29).

### Analysis of oxidized purine and pyrimidine bases of yeast DNA.

The alkaline comet assay was performed as described above. Slides were removed from the lysis solution and were washed three times in an enzyme buffer (40 mM HEPES, 100 mM KCl, 0.5 mM EDTA, 0.2 mg/ml bovine serum albumin, pH 8.0). Subsequently, they were drained and incubated with 70 μl formamidopyrimidine DNA-glycosylase (FPG) for 30 min at 37°C. Images of 100 randomly selected cells per group (50 cells from each of the two replicate slides) were visually analyzed. The number of oxidized purines (FPG-sensitive sites) was determined by subtracting the amount of strand breaks observed in the control samples (samples incubated only with buffer) from the total amount of breaks obtained after FPG incubation (27, 29).

### Annexin V staining.

Treated and untreated C. albicans cells were collected by centrifugation and digested with 2 mg/ml Zymolyase 20T (Seikagaku Corp., Japan) in potassium phosphate buffer (PPB) plus 1 M sorbitol (pH 6.0) for 2 h at 30°C. C. albicans protoplasts were stained with fluorescein isothiocyanate (FITC)-labeled annexin V and PI, using an FITC-annexin V apoptosis detection kit (Guava Nexin kit; Guava Technologies, Inc., Hayward, CA, USA). Subsequently, the cells were washed with PPB and incubated in an annexin binding buffer containing 5 μl/ml of FITC-annexin V and 5 μl of PI for 20 min. The cells were then analyzed by flow cytometry (Guava EasyCyte minisystem). For each experiment (*n* = 2), 10,000 events were evaluated, and cell debris was omitted from the analysis (29, 30).

### Biofilm viability.

Biofilm formation was performed according to the method of Pierce et al. (34), with modifications, using microtiter plates. For this test, Candida tropicalis strain 2 (see Table 1) incubated in yeast extract-peptone-dextrose at 35°C for 20 to 24 h was used. Then, the cells were collected by centrifugation at 3,000 × *g* for 5 min and washed twice with PBS buffer solution. The pellets were suspended one more time, and the cell density was adjusted to 1.0 × 10^6^ cells/ml in RPMI 1640 (Cultilab, São Paulo, Brazil). After 24 h, the wells were washed three times with PBS. Berberine was tested at concentrations ranging from 4.68 to 600 μg/ml. An aliquot of 200 μl of the drug solution was added to each well containing a viable 24-h-old biofilm. The plates were incubated at 35°C for 24 h. Measurement of the metabolic activity of the biofilm cells was evaluated using the 3-(4,5-dimethyl-2-thiazolyl)-2,5-diphenyl-2H-tetrazolium bromide (MTT; 1 mg/ml) colorimetric assay. Reading of the results was conducted in a microplate reader at 540 nm (35, 36).

### L929 cell cultivation.

L929 cells were cultivated under standard conditions in minimal essential medium with Earle's salts. All culture media were supplemented with 10% fetal bovine serum, 2 mM glutamine, 100 μg/ml penicillin, and 100 μg/ml streptomycin, and the cells were cultured at 37°C with 5% CO_2_. For assessment of cytotoxic effects, cells were grown for 2 days prior to treatment with the test substances. Afterwards, the medium was replaced with fresh medium containing the test substance or dimethyl sulfoxide (DMSO) solution as a control. The final DMSO concentration in the culture medium was kept constant at less than 0.1% (vol/vol).

### L929 cell proliferation inhibition using the MTT test.

Cell growth was quantified on the basis of the capacity of living cells to reduce the yellow dye MTT (Sigma-Aldrich) to the purple formazan product. V79 cells were plated in 96-well plates (0.3 × 10^6^ cells/well), and test compounds (0.039 to 25 μg/ml) dissolved in 0.1% DMSO were added to each well, followed by incubation for 24 h under standard cultivation conditions. Afterwards, the plates were centrifuged and the medium was replaced with fresh medium (150 μl) containing 0.5 mg/ml MTT. Three hours later, the MTT formazan product was dissolved in 150 μl DMSO and the absorbance was measured using a multiplate reader (Spectra Count; Packard, Canada). The effects of the test substances were quantified as the percentage of control absorbance of the reduced dye at 595 nm. Experiments were carried out in duplicate and repeated at least three times (29, 37).

### Statistical analysis.

*In vitro* susceptibility experiments were repeated at least three times on different days. Geometric means were used to compare the MIC results. The data obtained from the flow cytometry and alkaline comet assays were compared using one-way analysis of variance (ANOVA) followed by the Newman-Keuls test (*P* < 0.05). Mean values obtained from the assay of biofilm viability were analyzed by a parametric ANOVA, followed by the Tukey test (*P* < 0.05).

### Nucleotide sequence accession numbers.

The ITS/5.8S sequences were deposited in the GenBank database under accession numbers KJ740185, KJ740181, KJ740176, KJ740174, KJ740179, KJ740191, KJ740188, KJ740165, KJ740167, KJ740166, and KJ740168.

## RESULTS

### Molecular identification.

The entire ITS/5.8S region (ITS1, 5.8S, and ITS2) of the nuclear ribosomal DNA from yeast strains was amplified and sequenced, and the sequences were compared to sequences deposited in the GenBank database (data not shown). BLAST searches revealed that the sequences from the isolates were identical to the ITS/5.8S sequences from different isolates and C. albicans (*n* = 3), C. tropicalis (*n* = 2), C. parapsilosis (*n* = 2), and Cryptococcus neoformans (*n* = 4) strains, as shown in Table 1.

**TABLE 1:** Effects of berberine against FLC-resistant strains of Candida spp. and C. neoformans isolated in Ceara, Brazil

Strain	Origin	GenBank accession no.	MIC (μg/ml)	
			FLC	Berberine
Candida tropicalis 1	Blood	KJ740185	32	8
Candida tropicalis 2	Blood	KJ740181	16	8
Candida albicans 1	Blood	KJ740176	16	8
Candida albicans 2	Blood	KJ740174	32	8
Candida albicans 3	Blood	KJ740179	32	8
Candida parapsilosis 1	Blood	KJ740191	32	8
Candida parapsilosis 2	Blood	KJ740188	16	8
Cryptococcus neoformans 1	Blood	KJ740165	64	16
Cryptococcus neoformans 2	Blood	KJ740167	64	16
Cryptococcus neoformans 3	Blood	KJ740166	64	16
Cryptococcus neoformans 4	Urine	KJ740168	64	16
Candida krusei ATCC 6258			16	4
Candida parapsilosis ATTC 22019			1	16

### Berberine inhibits the growth of FLC-resistant Candida and Cryptococcus neoformans strains.

The fluconazole susceptibility profiles of the Candida and Cryptococcus neoformans strains were assessed by a microdilution technique, as previously described (23). Table 1 shows the variation in the susceptibility to fluconazole of the different strains tested, with the MICs ranging from 16 to 32 μg/ml for the Candida strains and with the MIC being 64 μg/ml for the Cryptococcus neoformans strains. All strains studied were inhibited by berberine to various degrees. Table 1 shows the potential antifungal activity of berberine against the yeast clinical isolates, for which the MICs ranged from 8 to 16 μg/ml. These results for berberine are promising. Therefore, it was decided to investigate the possible mechanism of action of berberine on Candida tropicalis strains using flow cytometric techniques.

### Berberine induces a loss of cell viability and plasma membrane damage in C. albicans.

**FIG 1:**
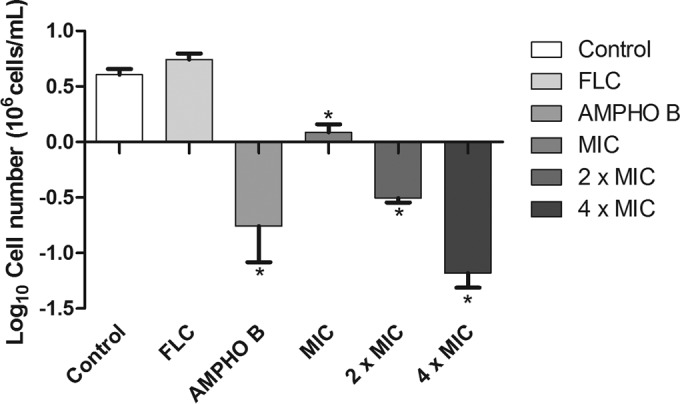
Effect of berberine on the number of viable cells of a C. albicans FLC-resistant strain. *, *P* < 0.05 compared to the results for the control, determined by ANOVA followed by the Newman-Keuls test.

**FIG 2:**
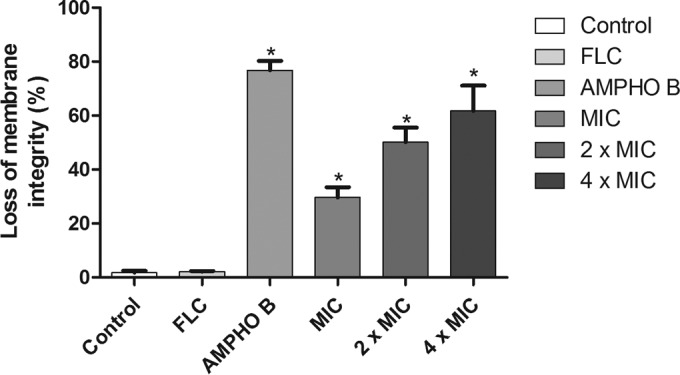
Effect of berberine at the MIC, 2× MIC, and 4× MIC on the stability of the cell membranes of the C. albicans FLC-resistant strains tested. *, *P* < 0.05 compared to the results for the control, determined by ANOVA followed by the Newman-Keuls test.

Berberine reduced the number of viable C. albicans cells at all concentrations tested in a concentration-dependent manner (Fig. 1). Moreover, the compound also promoted cell membrane instability in FLC-resistant yeast strains (Fig. 2). As shown in Fig. 1, exposure of the fluconazole-resistant strains to the azole did not cause a reduction in the number of viable cells compared to that for the control.

### Yeast Δψm changes are induced by berberine.

**FIG 3:**
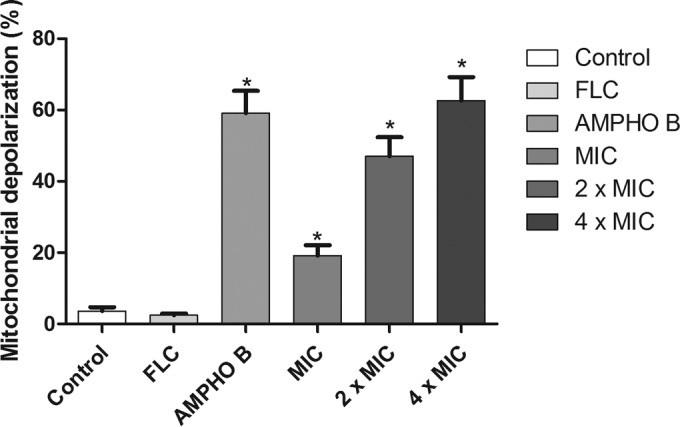
Assessment of the Δψm of fluconazole-resistant C. albicans strains. Cells were labeled with rhodamine 123 (50 nM). The graph shows the results for strains incubated for 24 h with RPMI 1640 (control), FLC (64 μg/ml), amphotericin B (4 μg/ml), and berberine at the MIC, 2× MIC, and 4× MIC. The percentage of cells of representative FLC-resistant Candida strains with mitochondrial dysfunction was evaluated for 24 h. *, *P* < 0.05 compared to the results for the control, determined by ANOVA followed by the Newman-Keuls test.

After 24 h of exposure to fluconazole, alterations of the yeast mitochondrial transmembrane potential (Δψm) were not observed in fluconazole-resistant strain C. albicans 2 (Fig. 3). In contrast, mitochondrial dysfunction was observed in a fluconazole-resistant C. albicans strain after treatment with berberine at several concentrations, suggesting that the treatment induced a reduction in the mitochondrial transmembrane potential (Fig. 3).

### Yeast cell phosphatidylserine externalization.

**FIG 4:**
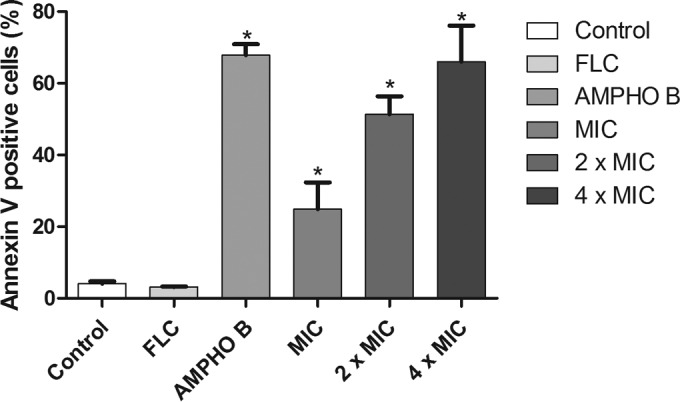
Phosphatidylserine externalization, which is observed at an early stage of apoptosis, shown by annexin V staining. The probe enabled the detection of alteration of phosphatidylserine localization from the inner membrane to the outer membrane. The fluorescence intensity indicates the amount of exposed phosphatidylserine in cells treated with berberine at the MIC, 2× MIC, and 4× MIC. The percentage of annexin V-positive cells of representative FLC-resistant C. albicans strains was evaluated for 24 h. *, *P* < 0.05 compared to the results for the control, determined by ANOVA followed by the Newman-Keuls test.

After 24 h of exposure, the percentage of cells with externalized phosphatidylserine after a single treatment with fluconazole was very close to that for the negative-control cultures (Fig. 4). After 24 h of incubation, yeast cultures treated with berberine at MIC, 2× MIC, and 4× MIC showed significant increases (*P* < 0.05) in the percentage of apoptotic cells compared to that for the control group: 22.64% ± 7.52%, 51.22% ± 5.02%, and 61.82% ± 10.04%, respectively.

### DNA damage.

**FIG 5:**
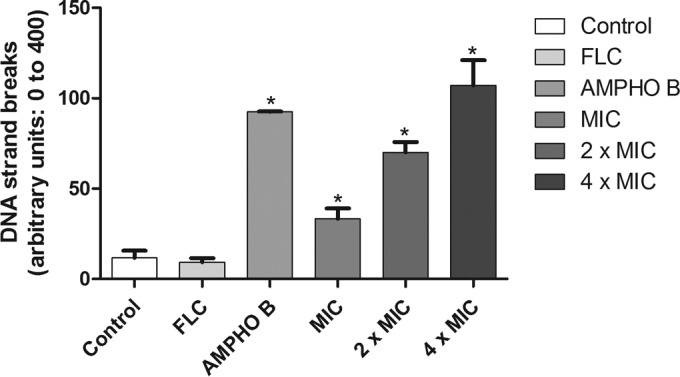
Effect of 24 h of incubation with RPMI 1640 (control), FLC (64 μg/ml), amphotericin B (4 μg/ml), and berberine at the MIC, 2× MIC, and 4× MIC on the DNA damage index. *, *P* < 0.05 compared to the results for the control, determined by ANOVA followed by the Newman-Keuls test.

**FIG 6:**
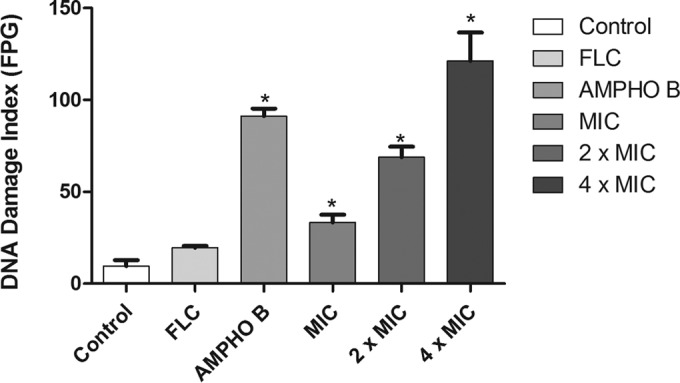
Effects of different treatments on the distribution of oxidative DNA damage in FLC-resistant C. albicans strains determined using the modified alkaline version (with FPG) of the comet assay. Yeasts were exposed to RPMI 1640 (control), FLC (64 μg/ml), amphotericin B (4 μg/ml), and berberine at the MIC, 2× MIC, and 4× MIC. *, *P* < 0.05 compared to the results for the control, determined by ANOVA followed by the Newman-Keuls test.

Figure 5 shows the amount of damage to the DNA of fluconazole-resistant C. albicans strains induced by berberine. Analysis of single cells for the levels of DNA damage (Fig. 5) showed that fluconazole induced low levels of DNA damage. In contrast, after 24 h of incubation, exposure of C. albicans to berberine resulted in a significant increase (*P* < 0.05) in DNA strand break levels (Fig. 5). Cells treated with berberine at the MIC, 2× MIC, and 4× MIC for 24 h exhibited damage index values (arbitrary units) of 32 ± 5.68, 71 ± 5.8, and 104 ± 14.11, respectively. Amphotericin B, used as a positive control, induced high DNA strand break levels. Furthermore, the compounds also promoted significant (*P* < 0.05) increases in the amounts of oxidized purine and pyrimidine (Fig. 6).

### Effect of berberine on the formed biofilm.

**FIG 7:**
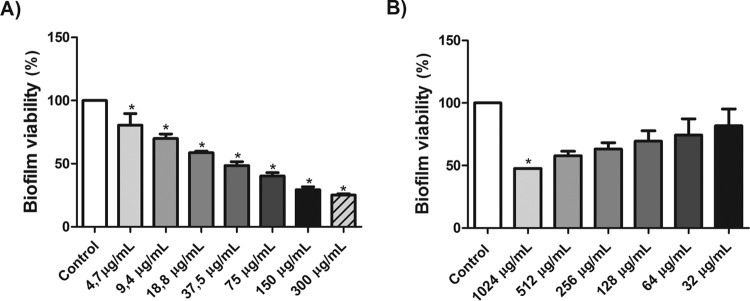
Effect of different concentrations of berberine (A) and fluconazole (B) on the metabolic activity of growing and mature biofilms of C. tropicalis, analyzed by the MTT reduction assay. *, *P* < 0.05 compared to the results for the control, determined by ANOVA followed by the Newman-Keuls test.

According to the results presented in Fig. 7, the berberine MIC for the C. tropicalis 2 isolate was less than 37.5 μg/ml. The antifungal effects of berberine caused a statistically significant reduction in the cellular activity of biofilm cells (*P* < 0.05), with the MIC being approximately four times higher than the MIC obtained with the same cells in the planktonic growth mode.

### Cytotoxic activity of berberine against mammalian L929 cells.

The cytotoxic activity of berberine against mammalian L929 cells was assessed. It is interesting to highlight that berberine showed low cytotoxicity, with the 95% confidence interval of the MIC being above 100 μg/ml in comparison to that for untreated cells (*P* < 0.05), as analyzed by the MTT assay.

## DISCUSSION

The results of this study show that berberine has antifungal activity against fluconazole-resistant Candida and Cryptococcus neoformans strains. According to some studies, berberine showed significant antifungal activity against Candida strains, which was consistent with the findings of this study (15, 38). An MIC of 16 μg/ml was obtained for the fluconazole-resistant Cryptococcus neoformans strains. Some studies that used berberine derivatives, such as *a*_9_-*O*-butyl-13-(4-isopropylbenzyl)berberine, have reported that berberine has antifungal activity against C. neoformans strains and obtained significant MICs (39, 40).

When strains were exposed to berberine in the presence of the cytofluorometric marker propidium iodide (PI), a decrease in the number of viable cells compared to that for the control was observed at the concentrations used for treatment (the MIC, 2× MIC, and 4× MIC). Thus, this finding indicates that berberine causes cell membrane damage and the possible impairment of cell function. The data corroborate those obtained by Dhamgaye et al. (15), who showed that berberine could cause a loss of membrane integrity, resulting in increased cell membrane permeability. Increased PI absorption in fluconazole-resistant Candida cells is an indication that berberine compounds promote cell death, considering that this marker can bind only to the nuclear DNA of dead cells (41).

The results of this study also indicate that cells treated with berberine promote Δψm changes, suggesting that berberine may affect the mitochondrial respiratory function, causing Δψm breakdown and the lack of accumulation of rhodamine 123 in the mitochondria (42). The collapse of Δψm may lead to transient pore openings in the membranes and the release of mitochondrial proapoptotic factors into the cytosol (43). Xu et al. (44) showed that treatment with fluconazole and berberine resulted in an increase in Δψm in fluconazole-resistant Candida albicans, suggesting that this increase is coupled with a low ATP level when ATP synthase activity is inhibited.

In the late stage of apoptosis, morphological changes, such as DNA fragmentation, are considered a late marker of this type of cell death (45). Thus, whether berberine triggers the apoptotic mechanism in fluconazole-resistant Candida albicans cells was investigated.

The alkaline version of the comet assay (standard protocol) is a sensitive procedure used to quantify different types of DNA damage to the cell, including single- and double-strand breaks (46). The results of this study showed that the DNA strands of cells treated with berberine at the MIC, 2× MIC, and 4× MIC showed total breaks, and it was found that the DNA strand breaks were more significant with increasing concentrations of berberine. In order to further assess the oxidative damage to DNA, the alkaline version of the comet assay was conducted in the presence of FPG (27, 47). The results of this study confirm that significant DNA damage, which is expressed as an index of DNA damage after treatment with the FPG enzyme compared to that for the negative control (untreated cells), is caused by treatment with berberine.

The increase in DNA damage was probably caused by the capacity of the FPG enzyme to recognize purine bases (adenine and guanine) within the DNA. Li et al. (14) demonstrated that berberine has a strong antifungal effect on C. albicans, causing cell cycle arrest and DNA damage. Other studies have also suggested that the berberine can bind to DNA, affecting DNA replication and transcription and the cell cycle (48, 49).

DNA condensation and fragmentation represent irreversible steps in cell death (43). The detection of apoptosis at an early stage can be determined using annexin V. In the presence of Ca^2+^, this marker binds with a high affinity to phosphatidylserine in apoptotic cell membranes (43). However, double staining with FITC-conjugated annexin V and PI allows discrimination between early apoptosis and necrosis (50). According to the data obtained in this study, berberine causes the death of fluconazole-resistant C. albicans cells by apoptosis.

Biofilm formation represents a major problem in hospitals, because, besides being able to form biomass in host tissue, Candida species can also grow on implanted medical devices in hospitalized patients (51). An important characteristic of Candida biofilms is resistance to antifungal agents, which can be intrinsic or acquired by the transfer of genetic material among biofilm cells (52), making new therapies necessary.

The results of this study showed that the berberine concentration necessary to inhibit both planktonic cells and preformed biofilm cells is similar. This indicates that berberine may reduce the growth of planktonic cells and inhibit the viability of cells in preformed biofilms at concentrations of 8 μg/ml and 37.5 μg/ml, respectively. This finding is relevant because biofilms are frequently associated with reduced sensitivity to conventional antifungal agents.

In tests of the cytotoxicity of berberine for mammalian L929 cells performed using the MTT assay, berberine showed low levels of cytotoxicity. The results of this study are in agreement with the findings of Gu et al. (53): berberine further altered the morphology of L929 cells only at concentrations greater than 100 μg/ml. Within this context, the antimicrobial activity and the low cytotoxic potential demonstrated by this compound reveal that it is a promising chemical compound for development as a new antimicrobial.

### Conclusion.

Studies related to the development of phytoproducts have been lacking, but this study has shown that treatment of fluconazole-resistant strains with one such phytoproduct, berberine, promoted alterations to the integrity of the plasma and mitochondrial membranes, possibly acting at specific sites near cell DNA, leading to death by apoptosis. The study also showed that berberine may reduce the viability of biofilms formed by fluconazole-resistant Candida tropicalis cells grown *in vitro*. Therefore, because of its antimicrobial activity, berberine is a promising source of molecules with antifungal properties.
